# Stability and Performance of Commercial Membranes in High-Temperature Organic Flow Batteries

**DOI:** 10.3390/membranes14080177

**Published:** 2024-08-15

**Authors:** Chiari J. Van Cauter, Yun Li, Sander Van Herck, Ivo F. J. Vankelecom

**Affiliations:** Membrane Technology Group (MTG), Division cMACS, Faculty Bioscience Engineering, KU Leuven, Celestijnenlaan 200F, P.O. Box 2454, 3001 Leuven, Belgium

**Keywords:** commercial ion exchange membrane, redox flow battery, extreme pH, performance screening, high temperature redox flow battery

## Abstract

Redox flow batteries (RFB) often operate at extreme pH conditions and may require cooling to prevent high temperatures. The stability of the battery membranes at these extreme pH-values at high temperatures is still largely unknown. In this paper, a systematic screening of the performance and stability of nine commercial membranes at pH 14 and pH ≤ 0 with temperatures up to 80 °C is conducted in an organic aqueous RFB. Swelling, area resistance, diffusion crossover, battery performance and membrane stability after 40–80 °C temperature treatment are shown, after which a recommendation is made for different user scenarios. The Aquivion E98-05 membrane performed best for both the Tiron/2,7-AQDS battery and the DHPS/Fe(CN)_6_ battery at 40 mA/cm^2^, with stable results after 1 week of storage at 80 °C. At 80 mA/cm^2^, E-620-PE performed best in the DHPS/Fe(CN)_6_ battery, while Sx-050DK performed best in the Tiron/2,7-AQDS battery.

## 1. Introduction

Adequate energy storage is an absolute necessity for the implementation of intermittent renewable energy. Lithium-ion batteries can only provide short-term storage, while redox flow batteries (RFBs) provide storage at much longer time scales. An additional benefit of RFBs is that changes in the electrode or in electrolyte tanks allow separate scaling of power and capacity. These advantages, along with their safety, make RFBs an ideal candidate for stationary storage.

In an RFB, the anode and cathode in the electrochemical cell are separated by a membrane. Electrolyte is pumped from separate containers to the anode and cathode. The membrane acts as a semipermeable barrier, separating the redox pairs and allowing passage of charge-balancing ions. As a result, membranes are one of the key materials in a battery, together with electrodes and electrolytes. A membrane with a high resistance to the passage of charge-balancing ions would result in low efficiency and a high IR drop. On the other hand, crossover of the redox pair would lower efficiency and capacity, leading to a reduced lifetime. The cost of the membrane is a further hindrance to the breakthrough of RFB technology. Cost estimations can go up to 60% of the total capital expenditure [1], depending on the exact type of RFB.

RFBs often operate at extreme pH conditions, i.e., pH ≤ 0 for many acid organic RFBs [2,3,4] and for vanadium RFBs [5], and pH 14 for many alkaline organic RFBs [6,7,8,9]. Recently, there is increased interest in using RFBs at higher temperatures [10,11,12]. The main restriction to date for using RFB at elevated temperature is the instability of the redox-active pair [11,13]. However, increased temperatures can also increase the activity of the electrolyte acid/base, making conditions even harsher on battery components. Nonetheless, all battery components must be stable at these extreme conditions to ensure successful operation. Membrane failure would be especially detrimental to battery performance. In particular, degradation of the membrane at extreme conditions could result in increased crossover, leading to capacity decay of the battery. Alternatively, degradation of the membrane could result in an altered ionic conductivity, limiting the battery’s cycling efficiency.

Commercial membranes, such as Nafion, are typically fluorine-based. They are used extensively and considered stable and performant in these extreme pH conditions [1,14,15,16,17]. Even though Nafion is currently considered as state-of-the-art, its high price can hamper the wide implementation of RFBs. Additionally, its fluorine-based chemistry is not aligned with the sustainable goals that e.g., the EU has set. This can be an extra barrier to the use of RFBs as a sustainable energy storage technology. To this end, other commercial membranes have been tested for their stability and performance in RFBs, where Nafion serves as a benchmark. A few examples of such commercially available alternative membranes are Aquivion [14], Fumasep FAP [15,18,19], VANADion [16,19], Aemion [20], Neosepta AFX [21], Neosepta AHA [17,19,21], Selemion AMV [19,21,22,23], Selemion ASV [21,23], Selemion DSV [21,23], and Selemion CMV [19]. Most research offers insight into the membrane performance for vanadium RFBs [14,15,16,17,18,19,20,22], with fewer focusing on pH-neutral organic flow batteries [21,23]. At elevated temperatures, most papers describe the use of Nafion in VRFBs with temperatures up to 45–50 °C [24,25] or up to 60 °C [13,26], with few reporting the use of Nafion in organic RFBs up to 40–55°C [27,28,29]. Fumasep membranes have been described in organic RFBs up to 60 °C [10,12,28] or as anion exchange membrane [30,31,32]. In contrast, the performance of commercial membranes at temperatures above 60 °C in RFBs has not yet been reported, nor is there extensive knowledge available on the stability of membranes at these elevated temperatures.

This paper compares the performance of nine cation exchange membranes (CEMs) in high alkalinity and high acidity and their stability in temperatures up to 80 °C, namely the Fumasep E-620, Fumasep E-620-PE, Aquivion E98-05S, Aquivion E98-05, Aquivion E98-09S, Selemion CMVN, FORBLUE S-2301WN, FORBLUE Sx-050DK, and FORBLUE Sx-053DK membranes.

As redox pairs, 7,8-dihydroxyphenazine-2-sulfonic acid (DHPS) and ferrocyanide/ferricyanide (FeCN) were used at pH 14. At pH 0, 1.2-dihydroxybenzene-3.5-disulfonic acid (Tiron) and anthraquinone-2,7-disulfonic acid disodium salt (2,7-AQDS) were used.

## 2. Materials and Methods

### 2.1. Materials

Table 1 gives an overview of the screened membranes, along with their IEM type, chemistry, and thickness. E-620 and E-620-PE were purchased from Fumatech (Bietigheim-Bissingen, Germany). CMVN was purchased from AGC Europe (Amsterdam, Netherlands; sourced in Japan). E98-05 and E98-09S were purchased through Sigma-Aldrich (Diegem, Belgium) and Merck Life Science (Hoeilaart, Belgium and Overijse, Belgium, respectively). E98-05S was purchased through Sigma-Aldrich (Diegem, Belgium). Sx-050DK, Sx-053DK and S-2301WN were purchased through AGC Europe (Amsterdam, Netherlands; sourced in Japan).

Potassium hydroxide (KOH, 99.98%) was purchased from Alfa Aesar (ThermoFischer, Kandel, Germany), KOH (90%) was purchased from Sigma-Aldrich (Sigma-Aldrich Chemie, Steinheim, Germany; made in France) sulfuric acid (H_2_SO_4_, 95–98%) was purchased from Acros Organics (Geel, Belgium), anthraquinone-2,7-disulfonic acid disodium salt (2,7-AQDS, >85%) was purchased from Chemcruz (Santa Cruz Biotechnology Inc, Dallas, TX, USA), and 1,2-dihydroxybenzene-3,5-disulfonic acid disodium salt monohydrate (Tiron, 97%) was purchased from Thermo Scientific (ThermoFischer, Kandel, Germany). DHPS and ferricyanide were provided by CMBlu Energy AG (Alzenau, Germany) in the context of the BALIHT project.

### 2.2. Methods

Water uptake (WU) and swelling ratio (SR) were measured to better understand the membrane transport, which heavily relies on the swelling-dependent inner structure of the membrane. To this extent, the mass, length, and thickness of wet and dry membranes were measured. For wet measurements, the membranes were stored in deionized water overnight and lightly dabbed with paper to remove surface water. For membranes CMVN, E98-05S, E98-05, E98-09S, Sx-050DK, Sx-053DK, and S-2301WN, dry measurements were measured as pristine membranes before wetting. For membranes E-620 and E-620-PE, dry measurements were measured after drying at 40 °C overnight, to account for leaching of the PEG additive. The WU and SR were calculated as follows:

WU = (W_wet_ − W_dry_)/W_dry_ × 100% (1)

SR, length = (L_wet_ − L_dry_)/L_dry_ × 100% (2)

SR, thickness = (T_wet_ − T_dry_)/T_dry_ × 100% (3)

where W_wet_, W_dry_, L_wet_, L_dry_, T_wet_, and T_dry_ represent the wet weight, dry weight, wet length, dry length, wet thickness and dry thickness, respectively. At least three samples were measured.

**Figure 1:**
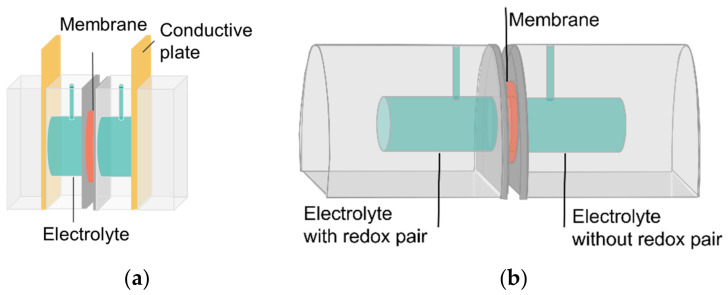
Scheme of the lab-made resistance cell (**a**) and diffusion cell (**b**).

The area resistance (AR) (ohm.cm^2^) was measured through potentiostatic electrochemical impedance spectroscopy (PEIS) with a BioLogic SAS SP-200 (Seyssinet-Pariset, France) over the range of 10–200 kHz and with a lab-made resistance cell (Figure 1a). The measurement was performed with only supporting electrolyte, i.e., 1M KOH or 1M H_2_SO_4_, or with supporting electrolyte and a membrane. The membranes were stored at least 2 days in the supporting electrolyte before measurement. The area resistance could then be calculated according to the following Equation (4):

AR = (R_2_ − R_1_) × A (4)

where R_1_ is the ohmic resistance of the cell containing only the supporting electrolyte, R_2_ is the ohmic resistance of the cell containing both the supporting electrolyte and the membrane, and A is the active membrane area being tested in the set-up. Thickness values stated by the manufacturer were used to calculate conductivity.

The membrane permeability for the redox-active molecules DHPS and Tiron were measured with a lab-made diffusion cell (Figure 1b). For the alkaline test, one side of the cell was filled with 0.1M DHPS in 1.6M KOH and with 1.8M KOH on the other side. For acid tests, 0.1M Tiron in 1M H_2_SO_4_ and 1.1M H_2_SO_4_ were used. Both sides were stirred continuously, and the diffusion of redox pairs was measured by taking 1 mL samples of the draw solution (KOH/H_2_SO_4_ side) at set time intervals. The volume was replenished with fresh KOH/H_2_SO_4_. This sample was analyzed by UV–VIS, and the dilution caused by the replenishments was taken into account via mass balances.

The permeability was calculated from the following Equation (5), according to Fick’s law:

(5) $$J=-D\frac{\partial C}{\partial x}↔D=\frac{-V\times T\times \ln\frac{C_{0}-C_{t}}{C_{0}}}{A\times \Delta t}$$

where V is the volume of each side of the diffusion cell, T is the thickness of the membrane, A is the area of the membrane, Δt is the time since the start of the experiment, C_0_ is the start concentration and C_t_ is the concentration at the time of measurement. Specifically, the volume was 15–17 mL, the thickness stated by the manufacturer was used, and the active membrane radius was 1 cm.

Selectivity is taken as the trade-off between area resistance and permeability. To this end, selectivity was compared between the different membranes as a plot of area resistance vs. permeability.

The chemical stability of the membranes was checked through attenuated total reflection–Fourier-transformed infrared (ATR-FTIR) (Bruker Alpha, Karlsruhe, Germany) before and after 1 week storage in 1M KOH and 1M H_2_SO_4_ at different temperatures. The membranes were washed with deionized water and dried overnight before ATR-FTIR measurement.

The morphology was studied through scanning electron microscopy (SEM) (JEOL JSM-6010LV, JEOL Europe, Zaventem, Belgium). The membranes were dried at 40 °C overnight, cryofractured in liquid nitrogen, and coated with Au/Pd conductive coating (JEOL JFC-1300 Auto Fine Coater, JEOL Europe, Zaventem, Belgium) before the measurements.

**Figure 2:**
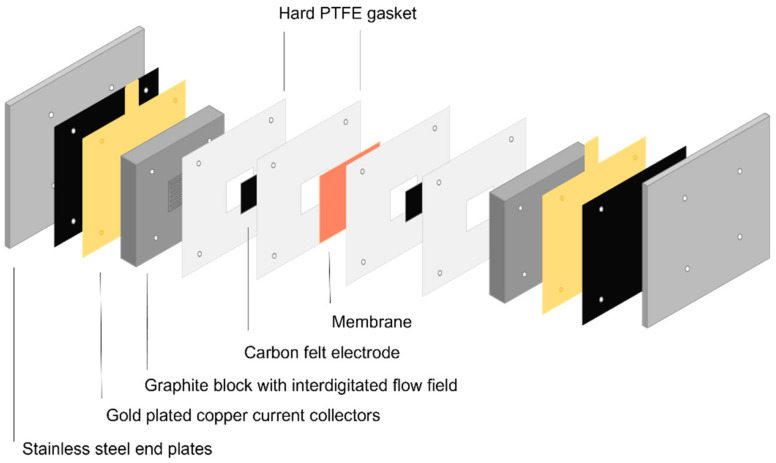
Scheme of the flow battery electrochemical cell. Adapted from [34].

Battery performance was tested with a Redox-flow.com A-cell, containing graphite blocks with 6.25 cm^2^ interdigitated flow field and PTFE gaskets (Figure 2). Every measurement was performed using a fresh carbon felt electrode (4 mm thickness, no pre-treatment) and fresh electrolyte. Energyscope software 3.7 was used. The measured current density was 40 mA/cm^2^ or 80 mA/cm^2^ and the pumping speed was 60 mL/min. For alkaline tests, 20 mL of 0.4 M DHPS in 1.6 M KOH and 20 mL of 0.8 M FeCN in 1.6 M KOH was used, provided by CMBlu. Cut-off voltages were set at 0.6 V and 1.6 V. For acid tests, 20 mL of 0.1M Tiron in 1M H_2_SO_4_ and 0.1M AQDS in 1M H_2_SO_4_ was used. Cut-off voltages were set at 0 V and 1 V. Both anolytes were bubbled with N_2_ gas for at least 1 h before the start of the measurement, as well as during the measurement. Membranes were soaked in 1M KOH or 1M H_2_SO_4_ overnight before testing.

## 3. Results and Discussion

### 3.1. Water Uptake and Swelling Ratio

Swelling of the membrane acts as an important preliminary indicator of both the resistance and the mechanical stability of the membrane. Many membranes rely on the concept of phase separation to obtain high conductivities, where the hydrophobic backbone and hydrophilic side chains self-assemble into separate domains [35]. The assembly of the hydrophilic side chains in clusters thus creates water transport channels [36]. These water channels are especially known in perfluorosulfonate-based membranes, such as Nafion or Aquivion. In this context, a high swelling degree is desired, as it leads to lower ohmic resistances. In terms of mechanical stability however, the opposite is true: a high swelling degree can cause mechanical stress and discrepancies in dimensions [19]. A subtle balance thus needs to be reached.

**Figure 3:**
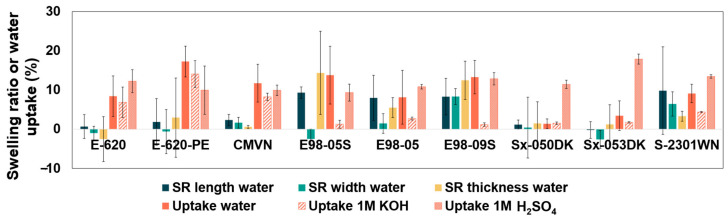
Swelling ratio (SR) per direction and water uptake (WU) for the selection of commercial membranes.

In Figure 3 and Tables S1 and S2, the swelling ratio, water uptake, and electrolyte uptake are depicted for each membrane, representing the dimensional changes and mass changes in the membrane upon wetting. It can be seen that the SPAEK-based E-620 and E-620-PE membranes show a negligible swelling ratio, even though the water uptake is not negligible. Aquivion membranes show one of the highest swelling ratios, and S-2301WN shows similar swelling and uptake behavior as Aquivion. Sx-050DK and Sx-053DK show limited swelling. High swelling of perfluorosulfonic membranes was hypothesized, as these membranes typically attain their high conductivity through their hydrophilic domains, with better conductivity at higher hydration levels [36]. Most membranes show a similar swelling behavior in both planar directions, except for E98-05S and Sx-053DK. It was expected that reinforcements, present in E-620-PE and S-2301WN, would restrict the swelling. However, this effect seems minimal in water when comparing the reinforced membranes with non-reinforced membranes of similar materials.

As all membranes were cation exchange membranes, a higher uptake of 1M H_2_SO_4_ was expected compared to uptake of 1M KOH. This behavior is clearly seen in the PFSA membranes, but not in the hydrocarbon membranes.

From these results, it is hypothesized that E-620-PE will have the lowest resistance in alkaline media, and Sx-050DK among the highest. In acid media, Sx-053DK is expected to have the lowest resistance.

A high standard deviation is present in the Fumasep membranes’ spatial swelling, indicating that these membranes might show some spatial inconsistencies. This inconsistency is likely due to their difficult handling, as the thin nature of these membranes causes a lot of curling.

In addition to the area resistance, swelling can also be a preliminary indicator for electrolyte diffusion. A high swelling degrees result in more open transport channels, which lead to high crossover. The order for crossover impact is expected to be opposite of the order of membrane resistance.

### 3.2. Area Resistance

The area resistance of the membrane is an important factor in the voltaic efficiency of the membrane, with high resistances leading to lower efficiencies due to increased ohmic losses. As mentioned earlier, many ion exchange membranes rely on phase separation to obtain high conductivities. In addition to the swelling, the thickness of the ion exchange membrane is a big determinant of its resistance. Thicker membranes result in a longer path for ion transport, making it increasingly difficult for ions to pass.

**Figure 4:**
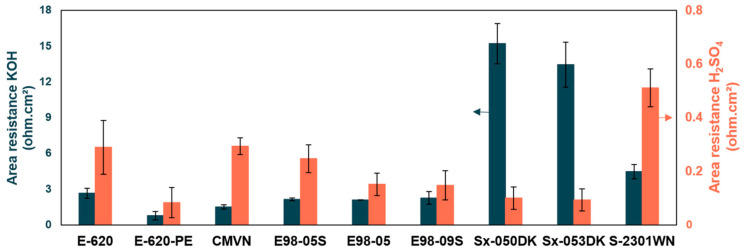
Area resistance of the membranes in 1M KOH (left axis, blue bar) and 1M H_2_SO_4_ (right axis, orange bar).

From the swelling behavior, it was hypothesized that E-620 and E98-09S would have a low resistance and Sx-050DK a high resistance. The latter showed the least swelling, so it is likely to have the smallest transport channels which could result in a high resistance. On the other hand, Fumasep membranes show among the highest water uptakes that, along with their thin nature, can lead to low resistances. As can be seen in Figure 4, this hypothesis was partly correct.

The order of area resistance in alkaline media is as follows: E-620-PE < CMVN < E98-05 < E98-05S < E98-09S < E-620 < S-2301WN < Sx-053DK < Sx-050DK. The order of area resistance in acid media is as follows: Sx-053DK ≤ E-620-PE ≤ Sx-050DK < E98-09S < E620 ≤ CMVN < E98-05 < E98-05S < S-2301WN. The ionic conductivity, normalized for membrane thickness, can be found in Figure S1. Comparison with values in the literature can be found in Table S4. Generally, a similar trend between the two media was expected, as both media rely on similar modes of transport, and hydrophilic channels will affect both media. On the other hand, the main charge carriers in alkaline and acid media have opposite charges. As a consequence, ion exchange groups present in the membrane can have opposite effects. It is, therefore, difficult to predict the trends in both media. Both of these effects seem present. It can be noted that the Sx-050DK and Sx-053DK membranes have the highest resistance in alkaline media and among the lowest resistance in acid media, thus having a clear affinity for protons.

Overall, resistances are significantly lower in acid media than in alkaline media. This difference can be explained by two factors. Firstly, a slightly higher ionic concentration is present in 1M H_2_SO_4_ compared to 1M KOH. A significant part of transport resistance in membranes stems from the used electrolyte. This is because three of the major ion transport mechanisms (diffusion, migration, and convection) take place through the interstitial phase of the membrane [35]. Additionally, the transport cluster size, shape and distribution inside the membrane are influenced by the supporting electrolyte [37]. Secondly, all tested membranes are CEMs, and, therefore, target the cations present in solution for transport. In acid media, protons are the main cations, while alkali metals, such as K^+^ in this work, are the main cations in alkaline media. K^+^ has a significantly larger Stokes radius than H^+^, leading to the intrinsically higher resistance of K^+^ transport compared to H^+^ transport. For H^+^ ions, the Grotthus mechanism also provides additional transport compared to K^+^ [35,36,38]. Additionally, K^+^ ions are known to dehydrate the ion exchange membrane, also leading to lower conductivities [36,39].

An inverse trend between area ohmic resistance and diffusion coefficient can usually be seen in membranes. Membranes with a low resistance generally have a more open structure and low resistance to ion transport, including redox-active species transport, leading to high diffusivities. Therefore, it is estimated that the Sx-050DK and Sx-053DK membranes will have a low diffusion coefficient in alkaline environment while the E-620-PE membrane will have the highest diffusion coefficient. In acid media, S-2301WN is hypothesized to have the lowest diffusion coefficient, while Sx-053DK is expected to have the highest.

### 3.3. Diffusion

The diffusion coefficient of active species in the membrane is an important factor in the coulombic efficiency of the membrane: a high permeability leads to lower coulombic efficiencies due to increased crossover and capacity losses. Permeability is typically influenced by the same factors as area resistance, but with opposite trends. High water uptake and swelling lead to high crossover of redox-active species. Thicker membranes have a longer path of ion transport, leading to a lower crossover.

**Figure 5:**
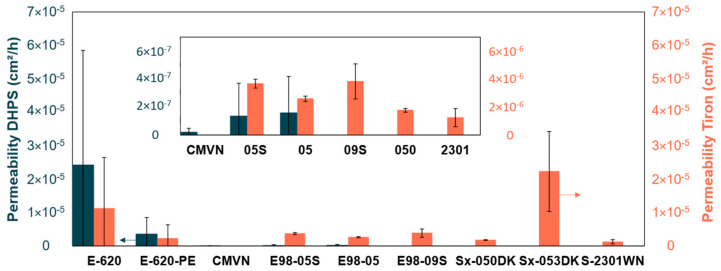
Diffusion coefficient of DHPS (left axis, blue bar) and Tiron (right axis, orange bar). The inset shows an enlarged depiction of smaller values.

Diffusion coefficients through the membrane for both DHPS in alkaline solution and Tiron in acid solution can be found in Figure 5. The average diffusion coefficient of DHPS in alkaline conditions decreases in the following order: E-620 > E-620-PE > E98-05 > E98-05S > CMVN > E98-09S = S-2301WN = Sx-050DK = Sx-053DK. However, standard deviations are high. The trade-off between area resistance and diffusion crossover can be seen in these results to some degree. The membranes with highest resistances (i.e., Sx-050DK, Sx-053DK and S-2301WN) showed negligible crossover. The membrane with the lowest resistance (E-620-PE) showed the second highest crossover. However, the trade-off trend is not seen clearly among the membranes E98-05S, E98-05, and CMVN, which showed similar resistances, or between E-620 and E-620-PE. Some correlation with swelling behavior, which influences both area resistance and diffusion crossover, can also be noted. Sx-050DK and Sx-053DK showed the lowest swelling behavior, coinciding with negligible crossover of DHPS.

The diffusion coefficient of Tiron in acid conditions decreases in the following order: Sx-053DK > E-620 > E98-09S ≥ E98-05S > E98-05 > E620-PE > Sx-050DK > S-2301WN > CMVN. It should be noted that E-620 and E-620-PE show large standard deviations. The trade-off between diffusion and area resistance is visible to some degree. The membrane with the highest area resistance, S-2301WN, has among the lowest Tiron crossover. However, the trade-off fails to explain some remarkable results, such as the large difference in diffusion between E-620 and E-620-PE, as well as the difference between Sx-050DK and Sx-053DK. The negligible crossover in CMVN can also not be related to the conductivity–selectivity trade-off. Among E98 membranes, E98-05 shows both the lowest area resistance and the lowest Tiron crossover, in contrast with the expected trade-off.

### 3.4. Battery Performance

Previously tested area resistances and diffusion coefficients through the membrane have an immense effect on the battery performance. A high area resistance can lead to a low VE, while high crossover can lead to low CE. Therefore, these criteria were used to select the best performing membranes for alkaline and acid media each, on which a battery cell test was subsequently performed. In alkaline media, E-620, E-620-PE, CMVN, E98-05S, E98-05, and E98-09S were tested. In acid media, E98-05, E98-09S, and Sx-050DK were tested. At increased current densities, higher CE and lower VE are hypothesized. This is because shorter cycling times lead to lower crossover, while increased current densities exacerbate the ohmic losses of the cell.

#### 3.4.1. Alkaline Battery Performance

**Figure 6:**
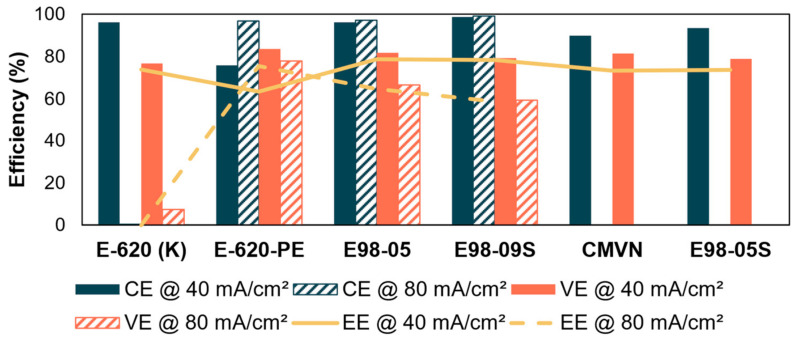
Efficiencies of different membranes in the DHPS/Fe(CN)_6_ battery at 40 mA/cm^2^ and 80 mA/cm^2^.

Based on the resistance and diffusion measurement, the best performing membranes were selected. In alkaline media, membranes with good overall selectivity as well as membranes with the lowest resistances were selected. The battery tests were performed with 0.4M DHPS and 0.8M Fe(CN)_6_ with carbon felt electrodes. At 40 mA/cm^2^, the membranes E-620, E-620-PE, CMVN, E98-05S, E98-05, and E98-09S had an initial EE of 73.8%, 63.3%, 73.1%, 73.6%, 78.6%, and 78.2%, respectively. At 80 mA/cm^2^, E-620, E-620-PE, E98-05, and E98-09S had an initial EE of 2.5%, 75.3%, 64.6%, and 58.7%, respectively. The results are shown in Figure 6 and Table S6.

From the area resistance results, it can be seen that E-620 has a lower VE than E-620-PE. This was expected, as the area resistance of E-620 was significantly higher than that of E-620-PE. However, comparison of CE between these two membranes does not follow a similar trend. The E-620-PE membrane also shows a slightly higher VE than other membranes, which is in accordance with its lowest area resistance.

E-620-PE had the lowest overall EE at 40 mA/cm^2^, with a value of ~63%. Other membranes show a similar EE with one another, with E98-05 and E98-09S having a slightly higher EE at approximately 78%.

Membranes with both best and worst efficiencies at 40 mA/cm^2^ were additionally tested at 80 mA/cm^2^. Typically, at higher current densities, crossover becomes less problematic due to shorter cycling times, resulting in higher CEs. At the same time, the increased current densities highlight the need for low resistance membranes, as high resistances can create a higher overpotential. This effect is clearly shown in the data: E-620-PE, which had both the best VE and the worst CE at 40 mA/cm^2^, performs much better at 80 mA/cm^2^, resulting in the best EE at 80 mA/cm^2^ of all tested membranes. In contrast, E-620 having the worst VE, showed a dramatically worsened battery cycling (Figure S2), in agreement with literature [28].

At the time of receipt of the DHPS electrolyte, the electrolyte was not yet optimized for extended cycling. Therefore, long term cycling stability is suboptimal, as capacity loss occurred after about six cycles (Figures S2–S7).

#### 3.4.2. Acid Battery Peformance

**Figure 7:**
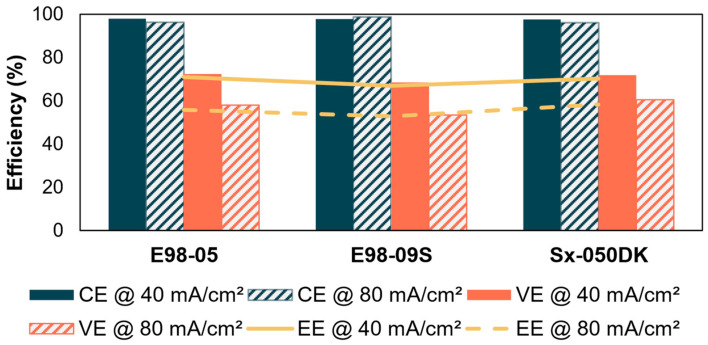
Efficiencies of different membranes in the Tiron/2,7-AQDS battery at 40 mA/cm^2^ and 80 mA/cm^2^.

Based on the resistance and diffusion measurement, the best performing membranes were selected. In acid media, membranes with good overall selectivity were selected. The battery tests were performed with 0.1M 2,7-AQDS and 0.1M Tiron with carbon–felt electrodes. At 40 mA/cm^2^, the membranes E98-05, E98-09S, and Sx-050DK had an initial EE of 70.9%, 67.0%, and 70.3%, respectively. At 80 mA/cm^2^, the EEs are 55.9%, 52.9%, and 58.3%, respectively. The results are shown in Figure 7 and Table S7. The low resistance of the membrane becomes increasingly important at higher current densities. Indeed, the low-resistance Sx-050DK obtained both the highest VE and highest EE.

Similar to the alkaline media battery tests, the electrolyte is not stable enough to test the membrane durability in extended cycling (Figures S8–S10 show cycling data). In the case of the Tiron/2,7-AQDS battery, instability has been reported with Tiron undergoing Michael addition, leading to capacity loss [2,40,41]. This can be mitigated by doubling the 2,7-AQDS volume and cycling as normal, or by replenishing 2,7-AQDS after the first cycle [2,41]. Both of these methods lead to the completion of side reactions on Tiron, after which regular cycling can be obtained with the extra/replenished 2,7-AQDS.

### 3.5. Stability to Extreme pH at High Temperature

The stability of the membrane is crucial for long-term battery operation, as chemical instability can influence the area resistance and/or the crossover negatively. The former will increase the IR-drop, while the latter will increase the crossover, impacting both the VE and CE, respectively. Multiple mechanisms could lead to the chemical instability of the membrane, such as the leaching of a component, degradation of ion exchange groups [42,43], or backbone degradation [42,43,44,45,46,47]. Other reasons for unstable operation could be limited mechanical stability [48] or the adsorption of active species to the membrane [43,49].

Increased temperatures can both increase the rate of degradation and facilitate new degradation paths that would not occur at room temperature. Therefore, testing membrane stability not only at room temperature but also at increased temperature is crucial to prove the stability.

#### 3.5.1. Alkaline Stability

The instability of the redox-active species does not allow for extended cycling stability, as a change in battery efficiency could not be directly linked to the membrane. Therefore, membrane stability tests are not performed through long-term battery operation but through proxies, such as area resistance (Figure 8, Table S8, thickness-normalized conductivities in Figure S11) and infrared spectroscopy (Figure 9 and Figures S13–S20).

**Figure 9:**
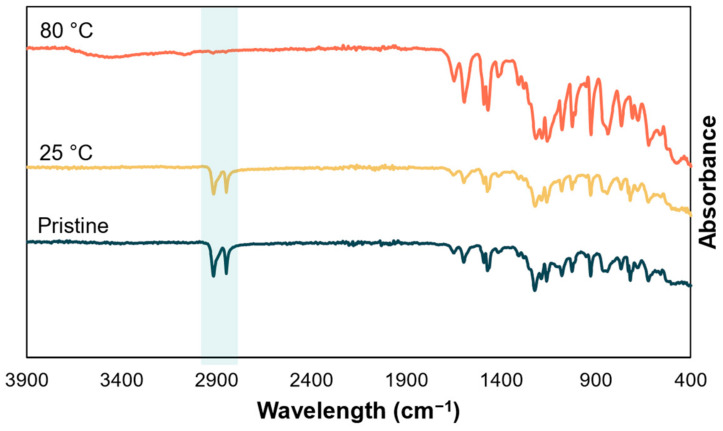
Infrared spectra of E-620 pristine and after 1 week storage in 1M KOH at 25 °C and 80 °C.

E98-05S, E98-05, and Sx-2301WN show stable area resistances with minimal or no changes at increased temperatures. Their infrared spectra show no significant changes. Therefore, these membranes are considered stable. E-620 shows increased resistance and large standard deviations after heating, already starting at 40 °C. Infrared spectra of E-620 (Figure 9) show the disappearance of the double peak at 2916 cm^−1^ and 2846 cm^−1^, corresponding to alkane C-H stretching. This is likely due to the removal of PEG, present in the membrane upon delivery. Use of E-620 is not recommended by the supplier beyond 40 °C, likely because of high swelling at these temperatures. E-620-PE shows deviating results at elevated temperatures compared to the as-received membranes. Use of E-620-PE is also not recommended by the supplier beyond 40 °C, likely because of high swelling at these temperatures. However, infrared spectra of E-620-PE (Figure S13a) show no significant changes.

CMVN shows an acceptable change in resistance data at 40 °C. At 60 °C, the resistance coincides with that of the as-received membrane, but high standard deviations are present. After 80°C treatment, the resistance dropped slightly. Infrared spectra (Figure S14a) show no significant changes. The supplier recommends a pH range below 7 and a temperature range up to 40 °C but mentions that the membrane can be used outside of this range. E98-09S shows increased resistances at elevated temperatures, starting from 40 °C, without a significant increase in standard deviation. At 80 °C, the resistance is within the range of the as-received membrane. The membrane is, thus, likely stable. Infrared spectra show no significant changes (Figure S17a). Sx-050DK has a high standard deviation at 40 °C but shows a resistance at 60 °C that is similar to the as-received membrane. Additionally, the infrared spectra (Figure S18a) show no significant changes, so the membrane is considered stable. However, the resistance after the 80 °C alkaline treatment is significantly lower. Sx-053DK shows a similar resistance at 40 °C compared to the as-received membrane, but slightly lowered resistance at 60 °C and drastically lower resistance after 80 °C. However, the membrane’s infrared spectra (Figure S19a) show no significant changes.

#### 3.5.2. Acid Stability

Just as in Section 3.5.1, proxies, such as area resistance (Figure 10, Table S9, thickness-normalized conductivities in Figure S21) and infrared spectroscopy (Figures S12–S20), are used to assess the membrane stability.

All tested membranes show stable area resistance with minimal or no changes at increased temperatures, and they show no significant changes in infrared spectra. It can be noted that E-620-PE shows large standard deviations after the testing procedure. At 80 °C, the area resistance becomes a fraction of the pristine value. This could be due to a lack of stability. The use of E-620-PE is not recommended by the supplier beyond 40 °C, so the low resistance is likely due to the high swelling of the membrane at such high temperatures. Instability at temperatures between 40 °C and 80 °C could not be concluded from these data due to the high standard deviations present. These standard deviations might also have occurred through different contents of PEG additive present in these membranes upon delivery and after leaching.

The area resistances at increased temperatures of E-620, E98-05, E98-09S, Sx-053DK, and Sx-2301WN all lie within the standard deviation of the as-received membrane, indicating no degradation. CMVN shows a slightly decreased area resistance at 80 °C, falling just below the standard deviation of the RT measurement. Furthermore, pH 5–7 is recommended for SELEMION membranes, but degradation of the membrane is only mentioned at high but not at low pH values. However, the infrared peaks show a slight change in relative intensity after extended storage in acid.

Sx-050DK also shows a slightly decreased area resistance at 80 °C, below the standard deviation of the as-received measurement. However, Sx-053DK has a similar chemical structure to that of Sx-050DK and shows no clear sign of degradation, leading to the conclusion that Sx-050DK is likely to be stable as well. Neither membrane shows a change in its infrared spectra. Similarly, E98-05S shows a clearly lowered resistance at all temperatures after storage in acid, but the E98-09S membrane with similar chemistry shows no sign of degradation. Neither membrane shows a change in its infrared spectra.

## 4. Conclusions

High-temperature operation of RFBs can be beneficial, if all components are stable in these conditions. However, membrane stability in high temperature at extreme pH has not been widely tested. Here, a systematic screening of a number of commercial membranes was performed for organic redox flow batteries at elevated temperatures. The commercial membranes E-620, E-620-PE, CMVN, E98-05S, E98-05, E98-09S, Sx-050DK, Sx-053DK, and S-2301WN were tested for water uptake and swelling, area resistance, diffusion of redox-active pairs through the membrane, battery cycling, and stability at temperatures up to 80 °C. In general, area resistances were lower in acid conditions compared to alkaline, and the diffusion of Tiron was higher than that of DHPS.

At pH 14, Fumasep E-620-PE had the lowest resistance (0.78 ohm.cm^2^). Membranes Aquivion E98-09S, FORBLUE Sx-050DK, FORBLUE Sx-053DK, and FORBLUE S-2301WN showed negligible crossover of redox-active species DHPS. Aquivion E98-05 showed the best energy efficiency at 40 mA/cm^2^ of 78.5%. Most membranes were chemically stable up to 80 °C in 1M KOH. Although no changes were visible in the infrared spectra of E-620-PE and Sx-053DK, performance declined at this elevated temperature.

At pH ≤ 0, FORBLUE Sx-053DK and E-620-PE had the lowest resistance at 0.08 ohm.cm^2^. CMVN showed negligible crossover of the redox-active species Tiron, while Aquivion E98-05 showed the best energy efficiency at 40 mA/cm^2^ of 71.0%. At 80 mA/cm^2^, Sx-050DK showed the best energy efficiency. All membranes were stable after 1 week in 1M H_2_SO_4_ at 80 °C.

Given the increased interest in redox flow batteries at elevated temperatures, it is recommended for authors to include these tests in their membrane optimization.

## Figures and Tables

**Figure 8:**
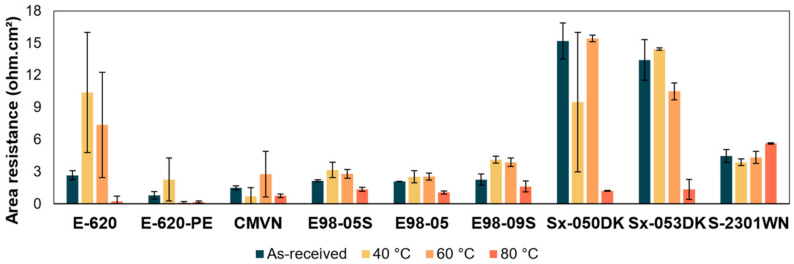
Area resistance of the as-received membranes and after 1 week storage in 1M KOH at 40 °C, 60 °C, and 80 °C.

**Figure 10:**
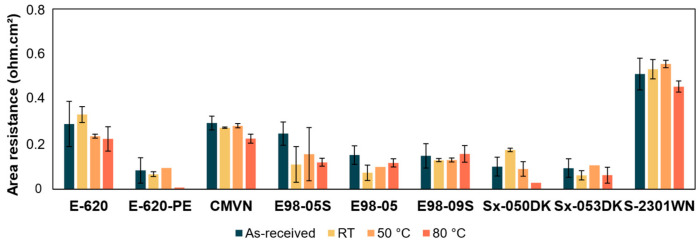
Area resistance before and after storage at RT, 50 °C, and 80 °C in 1M H_2_SO_4_ for 1 week.

**Table 1:** Overview of screened membranes and their characteristics.

Membrane	Company, Series	Type	Counter ion	Chemistry	Thickness (µm)
E-620	Fumatech, fumasep	CEM	K^+^	Sulfonated poly aryl ether ketone (SPAEK) [33]	20
E-620-PE	Fumatech, fumasep	CEM	K^+^	SPAEK [33] with poly ethylene (PE) reinforcement	20
CMVN	AGC, Selemion	CEM	Na^+^	Hydrocarbon with polyolefin reinforcement	100
E98-05S	Solvay, Aquivion	CEM	H^+^	Perfluorinated sulfonic acid	50
E98-05	Solvay, Aquivion	CEM	H^+^	Perfluorinated sulfonic acid	50
E98-09S	Solvay, Aquivion	CEM	H^+^	Perfluorinated sulfonic acid	90
Sx-050DK	AGC, FORBLUE	CEM	K^+^	Perfluorinated sulfonic acid	50
Sx-053DK	AGC, FORBLUE	CEM	K^+^	Perfluorinated sulfonic acid	50
S-2301WN	AGC, FORBLUE	CEM	Na^+^	Perfluorinated sulfonic acid with poly tetra fluoroethylene (PTFE) fiber reinforcement	330

## Data Availability

Data is contained within the article or Supplementary Information.

## Supplementary Materials

The following supporting information can be downloaded at: https://www.mdpi.com/article/10.3390/membranes14080177/s1, Table S1: Water swelling ratio (SR) for the tested commercial membranes, Table S2: Water and electrolyte uptake for the tested commercial membranes, Table S3: Area resistances of the tested commercial membranes in both alkaline and acid environments, Table S4: Comparison of area resistance or ionic conductivity of the tested commercial membranes to the literature, Table S5: Diffusion coefficients of the tested commercial membranes for DHPS and Tiron, Table S6: Efficiencies of different membranes in DHPS/FeCN battery at 40 mA/cm^2^ and 80 mA/cm^2^, Table S7: Efficiencies of different membranes in 2,7-AQDS/Tiron battery at 40 mA/cm^2^ and 80 mA/cm^2^, Table S8: Area resistance before and after storage in 40 °C, 60 °C, and 80 °C 1M KOH for 1 week, Table S9: Area resistance before and after storage in RT, 50 °C, and 80 °C 1M H_2_SO_4_ for 1 week, Figure S1: Ionic conductivity of the membranes in 1M KOH and 1M H_2_SO_4_, Figure S2: cycling efficiencies of DHPS/FeCN battery with E-620 membrane at 40 mA/cm^2^ (a) and 80 mA/cm^2^ (b) and voltage curve at 80 mA/cm^2^ (c), Figure S3: Cycling efficiencies of DHPS/FeCN battery with E-620-Pe membrane at 40 mA/cm^2^ (a) and 80 mA/cm^2^ (b), Figure S4: Cycling efficiencies of DHPS/FeCN battery with E98-05 membrane at 40 mA/cm^2^ (a) and 80 mA/cm^2^ (b), Figure S5: Cycling efficiencies of DHPS/FeCN battery with E98-09S membrane at 40 mA/cm^2^ (a) and 80 mA/cm^2^ (b). Figure S6: Cycling efficiencies of DHPS/FeCN battery with CMVN membrane at 40 mA/cm^2^, Figure S7: Cyclin efficiencies of DHPS/FeCN battery with E98-05S membrane at 40 mA/cm^2^, Figure S8: Cycling efficiencies of Tiron/2,7-AQDS battery with E98-05 membrane at 40 mA/cm^2^ (a) and 80 mA/cm^2^ (b), Figure S9: Cycling efficiencies of Tiron/2,7-AQDS battery with E98-09S membrane at 40 mA/cm^2^ (a) and 80 mA/cm^2^ (b), Figure S10: Cycling efficiencies of Tiron/2,7-AQDS with Sx-050DK membrane at 40 mA/cm^2^ (a) and 80 mA/cm^2^ (b), Figure S11: Ionic conductivity of the as-received membranes and after 1 week storage in 1M KOH at 40 °C, 60 °C, and 80 °C, Figure S12: ATR-FTIR spectra of E-620 after 1 week 1M H_2_SO_4_ treatment at different temperatures, Figure S13: ATR-FTIR spectra of E-620-PE after 1 week 1M KOH treatment at different temperatures (a) and 1 week 1M H_2_SO_4_ treatment at different temperatures (b), Figure S14: ATR-FTIR spectra of CMVN after 1 week 1M KOH treatment at different temperatures (a) and after 1 week H_2_SO_4_ treatment at different temperatures plus 42.5 weeks at room temperature (b), Figure S15: ATR-FTIR spectra of E98-05S after 1 week 1M KOH treatment at different temperatures (a) and 1 week 1M H_2_SO_4_ treatment at different temperatures (b), Figure S16: ATR-FTIR spectra of E98-05 after 1 week 1M KOH treatment at different temperatures (a) and 1 week 1M H_2_SO_4_ treatment at different temperatures (b), Figure S17: ATR-FTIR spectra of E98-09S after 1 week 1M KOH treatment at different temperatures (a) and 1 week 1M H_2_SO_4_ treatment at different temperatures (b), Figure S18: ATR-FTIR spectra of Sx-050DK after 1 week 1M KOH treatment at different temperatures (a) and 1 week 1M H_2_SO_4_ treatment at different temperatures (b), Figure S19: ATR-FTIR spectra of Sx-053DK after 1 week 1M KOH treatment at different temperatures (a) and 1 week 1M H_2_SO_4_ treatment at different temperatures (b), Figure S20: ATR-FTIR spectra of S-2301WN after 1 week 1M KOH treatment at different temperatures (a) and 1 week 1M H_2_SO_4_ treatment at different temperatures plus 42.5 weeks at room temperature (b), Figure S21: Ionic conductivities before and after storage at RT, 50 °C, and 80 °C in 1M H_2_SO_4_ for 1 week, Figure S22: SEM pictures of E-620 (a), E-620-PE (b), CMVN (c), E98-05S (d), E98-05 (e), E98-09S (f), Sx-050DK (g), Sx-053DK (h), and S-2301WN (i).
