# Supplementary material for: Stability and Performance of Commercial Membranes in High-Temperature Organic Flow Batteries
Source: Membranes (Basel). 2024 Aug 15;14(8):177. doi: 10.3390/membranes14080177 (PMC11356106; doi:10.3390/membranes14080177)
Supplement: Supplementary file 1 [file membranes-14-00177-s001.zip › membranes-3048728-supplementary.pdf]

Supporting information

Table S1: Water swelling ratio (SR) for the tested commercial membranes.

Membrane	SR length (%)	SR width (%)	SR thickness (%)
E-620	0.7 ± 3.1	-1.0 ± 1.7	-2.5 ± 5.7
E-620-PE	1.8 ± 5.9	-0.6 ± 5.6	2.9 ± 10.1
CMVN	2.33 ± 1.4	1.6 ± 1.4	0.6 ± 0.4
E98-05S	9.3 ± 1.5	-2.5 ± 2.5	14.3 ± 10.6
E98-05	8.0 ± 5.7	1.4 ± 2.5	5.5 ± 2.5
E98-09S	8.3 ± 4.7	8.3 ± 2.0	12.5 ± 4.9
Sx-050DK	1.2 ± 1.2	0.4 ± 7.7	1.5 ± 5.5
Sx-053DK	-0.2 ± 2.1	-2.6 ± 2.3	1.2 ± 5.0
S-2301WN	9.8 ± 11.2	6.4 ± 3.1	3.3 ± 1.3

Table S2: Water and electrolyte uptake for the tested commercial membranes.

Membrane	Water uptake (%)	1M KOH uptake (%)	1M H_2SO_4 uptake (%)
E-620 (K)	8.4 ± 5.2	6.8 ± 3.9	12.2 ± 2.9
E-620-PE	17.3 ± 3.9	14.1 ± 3.4	9.9 ± 6.2
CMVN	11.7 ± 4.8	8.2 ± 0.9	9.9 ± 1.3
E98-05S	13.8 ± 7.4	1.2 ± 1.0	9.3 ± 2.2
E98-05	8.1 ± 6.9	2.7 ± 0.4	10.8 ± 0.6
E98-09S	13.3 ± 4.3	1.2 ± 0.4	12.8 ± 1.6
Sx-050DK	1.4 ± 1.2	1.5 ± 0.3	11.4 ± 1.1
Sx-053DK	3.4 ± 3.8	1.7 ± 0.2	17.9 ± 1.3
S-2301WN	9.1 ± 2.4	4.4 ± 0.1	13.4 ± 0.4

Table S3: Area resistances of the tested commercial membranes in both alkaline and acid environment.

	Area resistance (ohm.cm²)	
**Membrane**	**1M KOH**	**1M H_2SO_4**
E-620(K)	2.66 ± 0.42	0.29 ± 0.10
E-620-PE	0.78 ± 0.35	0.08 ± 0.06
CMVN	1.51 ± 0.18	0.29 ± 0.03
E98-05S	2.15 ± 0.11	0.24 ± 0.05
E98-05	2.10 ± 0.01	0.15 ± 0.04
E98-09S	2.26 ± 0.53	0.15 ± 0.05
Sx-050DK	15.21 ± 1.69	0.10 ± 0.04
Sx-053DK	13.44 ± 1.89	0.09 ± 0.04
S-2301WN	4.47 ± 0.59	0.51 ± 0.07

Table S4: Comparison of area resistance or ionic conductivity of the tested commercial membranes to literature

Membrane	This work	Others' work
E-620(K)	1M KOH<br>2.66 ± 0.42 ohm.cm²	1M KOH<br>0.74 ohm.cm²<br>[1]
CMVN	1M H_2SO_4<br>34.3 ± 3.6 mS/cm	2.5M H_2SO_4<br>38.1 mS/cm<br>[2]

1. Wang, C.; Yu, B.; Liu, Y.; Wang, H.; Zhang, Z.; Xie, C.; Li, X.; Zhang, H.; Jin, Z. N-Alkyl-Carboxylate-Functionalized Anthraquinone for Long-Cycling Aqueous Redox Flow Batteries. *Energy Storage Mater.* **2021**, *36*, 417–426, doi:10.1016/j.ensm.2021.01.019.

2. Düerkop, D.; Widdecke, H.; Kunz, U.; Schilde, C.; Schiemann, A. Low-Cost Membranen Für Die Vanadium-Redox-Flow-Batterie. *Chemie Ing. Tech.* **2021**, *93*, 1445–1450, doi:10.1002/cite.202100033.

Table S5: Diffusion coefficients of the tested commercial membranes for DHPS and Tiron.

**Membrane**	**Diffusion coefficient [10^{-8} cm²/h], DHPS**	**Diffusion coefficient [10^{-6} cm²/h], Tiron**
E-620(K)	2425 ± 3428	11.22 ± 15.21
E-620-PE	354.9 ± 493.2	2.249 ± 4.110
CMVN	1.973 ± 2.791	Negligible
E98-05S	13.52 ± 23.39	3.671 ± 0.3179
E98-05	15.80 ± 26.03	2.580 ± 0.1895
E98-09S	Negligible	3.827 ± 1.252
Sx-050DK	Negligible	1.762 ± 0.1362
Sx-053DK	Negligible	22.27 ± 11.97
S-2301WN	Negligible	1.232 ± 0.6431

Table S6: Efficiencies of different membranes in DHPS/Fe(CN)6 battery at 40mA/cm² and 80mA/cm².

**Membrane**	**Current density (mA/cm²)**	**CE (%)**	**VE (%)**	**EE (%)**
E-620 (K)	40	96.2	76.7	73.8
	80	0.3	7.5	0.025
E-620-PE	40	75.8	83.5	63.3
	80	96.7	77.9	75.3
CMVN	40	89.6	81.4	73.1
E98-05S	40	93.2	79.2	73.8
E98-05	40	96.2	81.7	78.5
	80	97.2	66.4	64.6
E98-09S	40	98.7	79.2	78.2
	80	99.2	59.2	58.7

Table S7: Efficiencies of different membranes in 2,7-AQDS/Tiron battery at 40mA/cm² and 80mA/cm².

Membrane	Current density (mA/cm²)	CE (%)	VE (%)	EE (%)
E98-05	40	98.0	72.4	71.0
	80	96.4	58.0	55.9
E98-09S	40	97.8	68.5	67.0
	80	98.7	53.5	52.9
Sx-050DK	40	97.7	72.0	70.3
	80	96.2	60.6	58.3

Table S8: Area resistance (ohm.cm²) before and after storage in 40°C, 60°C and 80°C 1M KOH for 1 week.

	As-received	40°C	60°C	80°C
**E-620 (K)**	2.66 ± 0.42	10.40 ± 5.62	7.37 ± 4.91	0.23 ± 0.48
**E-620-PE**	0.78 ± 0.35	2.27 ± 2.00	0.12 ± 0.10	0.17 ± 0.10
**CMVN**	1.51 ± 0.18	0.71 ± 0.80	2.77 ± 2.14	0.75 ± 0.16
**E98-05S**	2.15 ± 0.11	3.17 ± 0.73	2.80 ± 0.40	1.36 ± 0.18
**E98-05**	2.10 ± 0.01	2.52 ± 0.57	2.55 ± 0.32	1.06 ± 0.13
**E98-09S**	2.26 ± 0.53	4.12 ± 0.32	3.89 ± 0.40	1.62 ± 0.52
**Sx-050DK**	15.21 ± 1.69	9.50 ± 6.51	15.45 ± 0.31	1.22 ± 0.05
**Sx-053DK**	13.44 ± 1.89	14.46 ± 0.10	10.51 ± 0.79	1.34 ± 0.94
**S-2301WN**	4.47 ± 0.59	3.88 ± 0.31	4.33 ± 0.56	5.65 ± 0.05

Table S9: Area resistance (ohm.cm²) before and after storage in RT, 50°C and 80°C 1M H_2SO_4 for 1 week.

	As-received	RT	50°C	80°C
**E-620 (K)**	0.29 ± 0.10	0.33 ± 0.04	0.24 ± 0.01	0.22 ± 0.05
**E-620-PE**	0.08 ± 0.06	0.07 ± 0.01	0.09	0.01
**CMVN**	0.29 ± 0.03	0.27 ± 0.00	0.28 ± 0.01	0.22 ± 0.02
**E98-05S**	0.25 ± 0.05	0.11 ± 0.08	0.16 ± 0.12	0.12 ± 0.02
**E98-05**	0.15 ± 0.04	0.07 ± 0.03	0.09	0.12 ± 0.02
**E98-09S**	0.15 ± 0.05	0.13 ± 0.01	0.13 ± 0.01	0.16 ± 0.04
**Sx-050DK**	0.10 ± 0.04	0.17 ± 0.01	0.09 ± 0.03	0.03
**Sx-053DK**	0.09 ± 0.04	0.06 ± 0.02	0.11	0.06 ± 0.04
**S-2301WN**	0.51 ± 0.07	0.53 ± 0.04	0.56 ± 0.02	0.46 ± 0.02

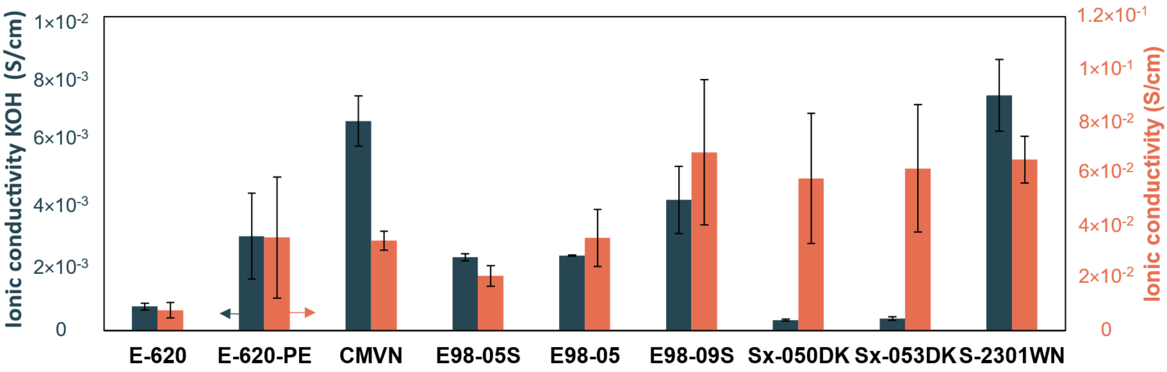

Figure S1: Ionic conductivity of the membranes in 1M KOH (left axis, blue bar) and 1M H_2SO_4 (right axis, orange bar)

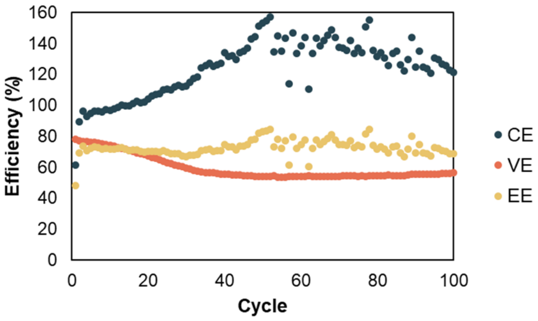

a.

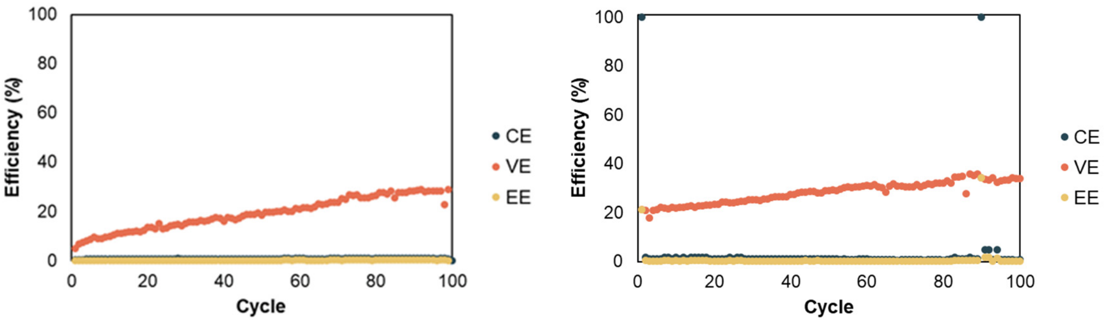

b.

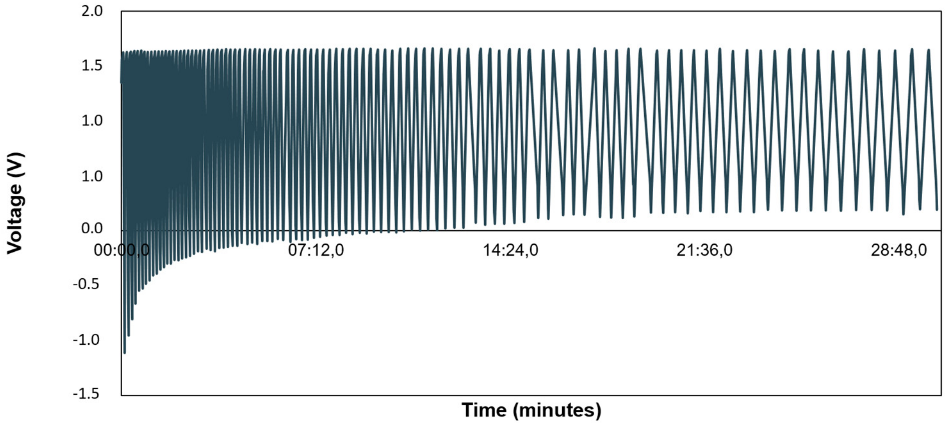

c.

Figure S2: Cycling efficiencies of DHPS/FeCN battery with E-620 membrane at 40mA/cm² (a) and 80mA/cm² (b) and voltage curve at 80mA/cm² (c). 80mA/cm² shows 2 subsequent cyling, with unchanged electrode and electrolyte. The voltage curve of the first 100 cycli at 80mA/cm² is shown in figure c.

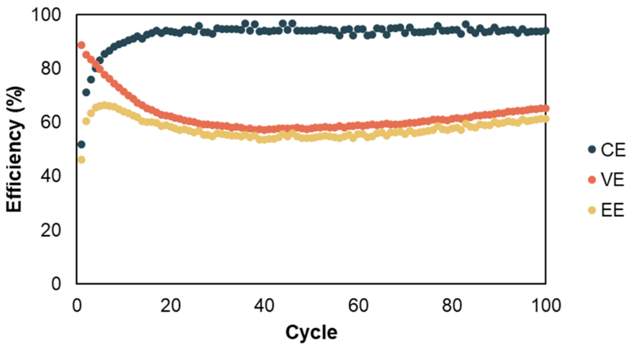

a.

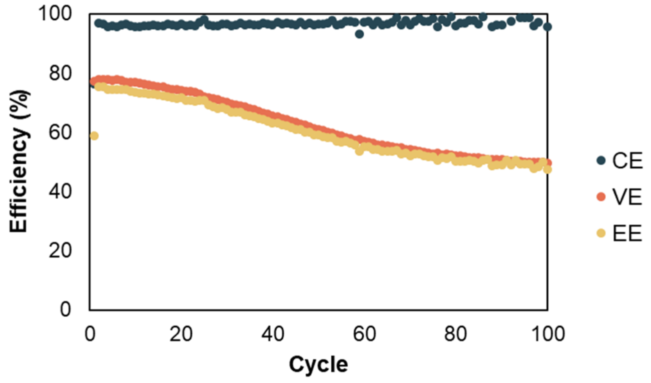

b.

Figure S3: Cycling efficiencies of DHPS/FeCN battery with E-620-PE membrane at 40mA/cm² (a) and 80mA/cm² (b).

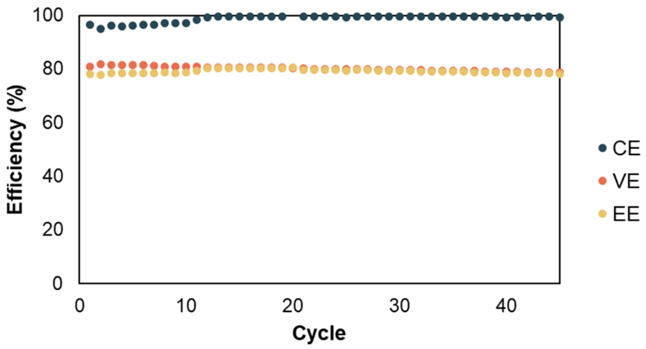

a.

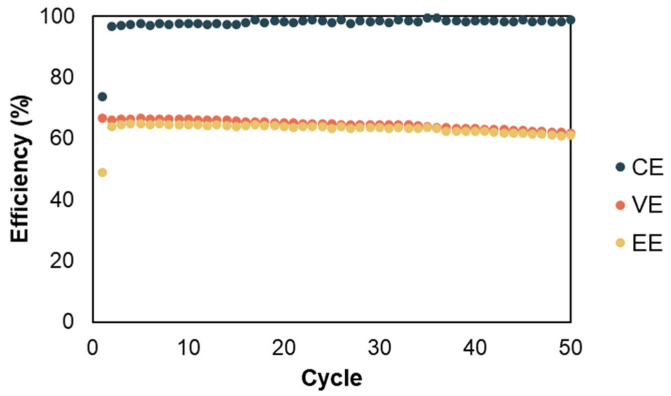

b.

Figure S4: Cycling efficiencies of DHPS/FeCN battery with E98-05 membrane at 40mA/cm² (a) and 80mA/cm² (b).

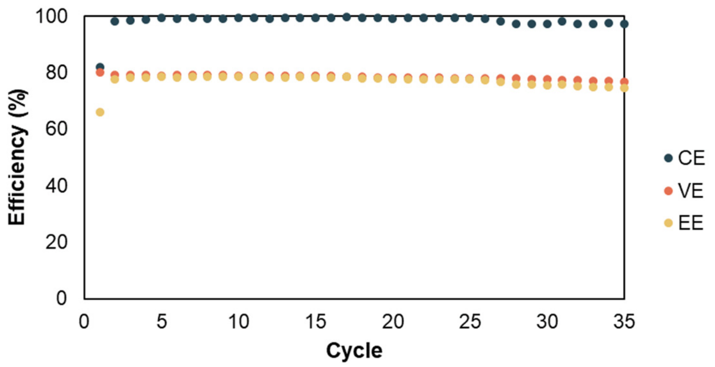

a.

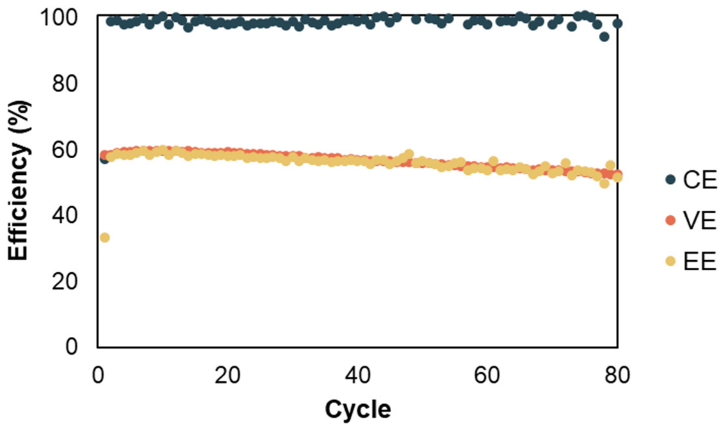

b.

Figure S5: Cycling efficiencies of DHPS/FeCN battery with E98-09S membrane at 40mA/cm² (a) and 80mA/cm² (b).

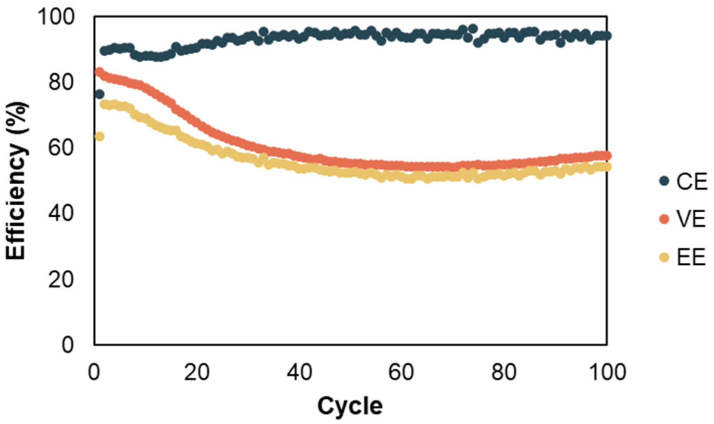

Figure S6: Cycling efficiencies of DHPS/FeCN battery with CMVN membrane at 40mA/cm².

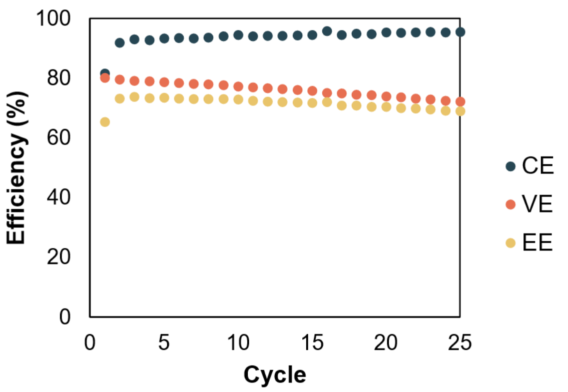

Figure S7: Cycling efficiencies of DHPS/FeCN battery with E98-05S membrane at 40mA/cm².

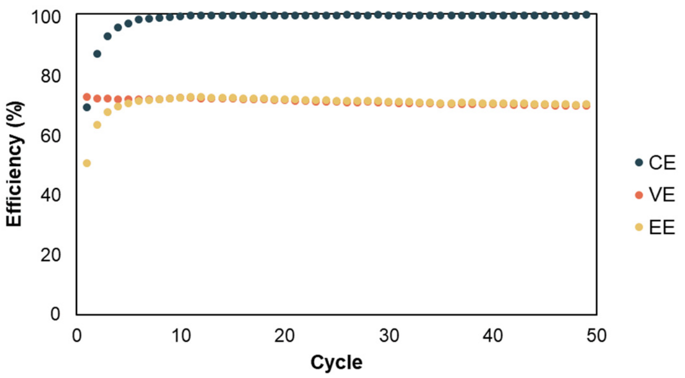

a.

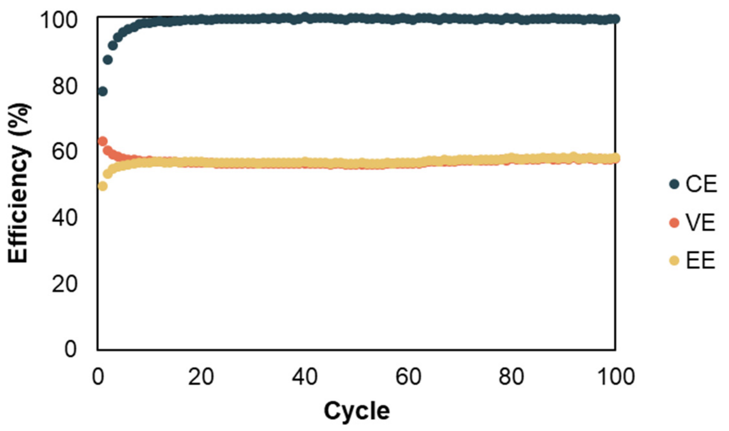

b.

Figure S8: Cycling efficiencies of Tiron/2,7-AQDS battery with E98-05 membrane at 40mA/cm² (a) and 80mA/cm² (b).

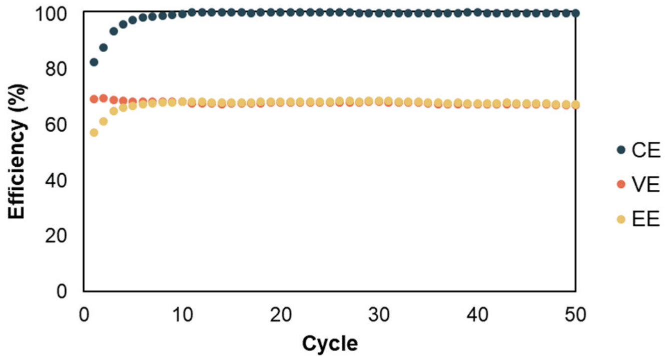

a.

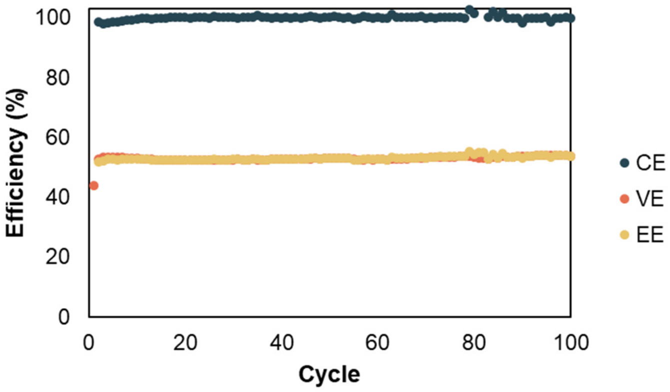

b.

Figure S9: Cycling efficiencies of Tiron/2,7-AQDS battery with E98-09S membrane at 40mA/cm² (a) and 80mA/cm² (b).

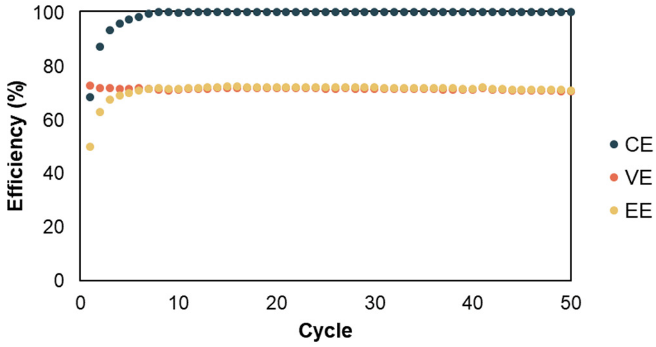

a.

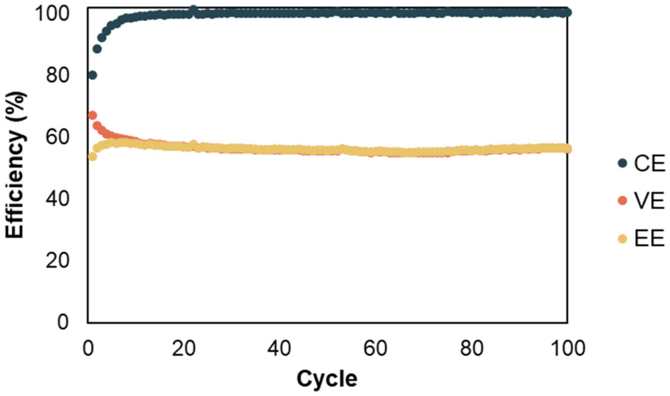

b.

Figure S10: Cycling efficiencies of Tiron/2,7-AQDS battery with Sx-050DK membrane at 40mA/cm² (a) and 80mA/cm² (b).

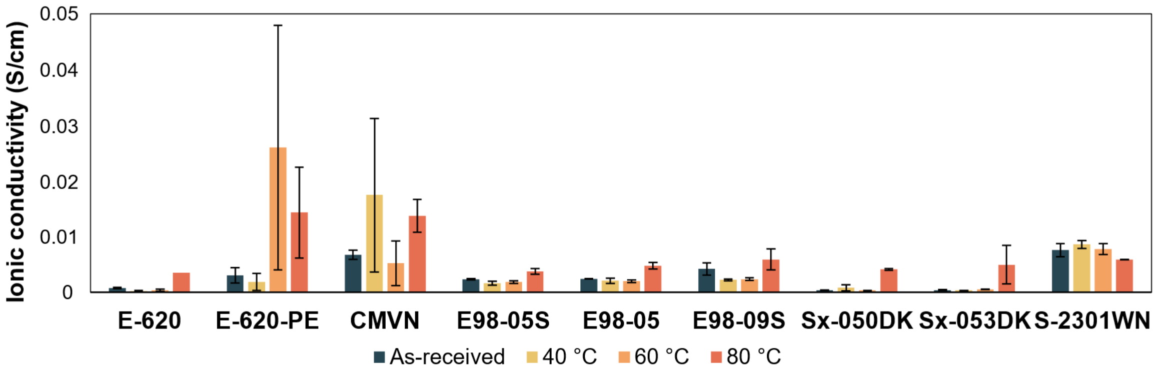

Figure S11: Ionic conductivity of the as-received membranes and after 1 week storage in 1M KOH at 40 °C, 60 °C and 80 °C.

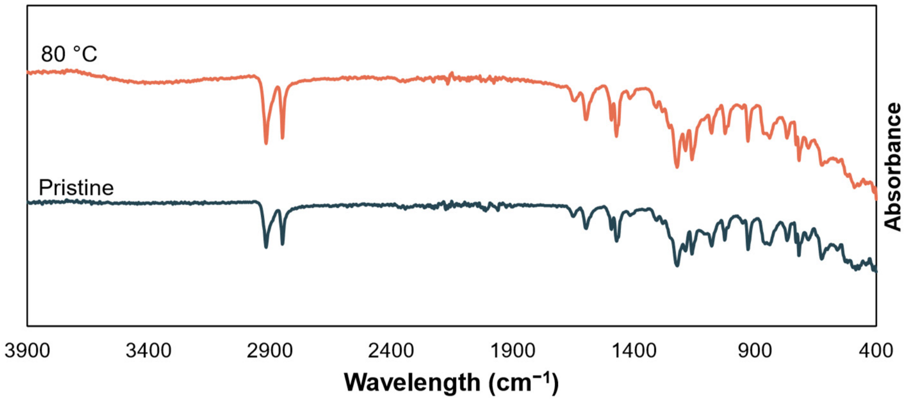

Figure S12: ATR-FTIR spectra of E-620 after 1 week 1M H_2SO_4 treatment at different temperatures.

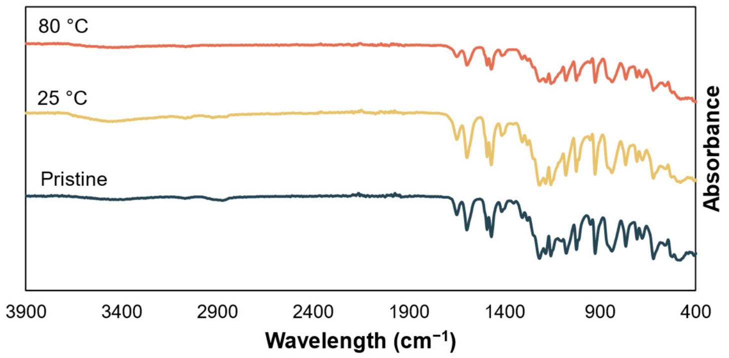

a.

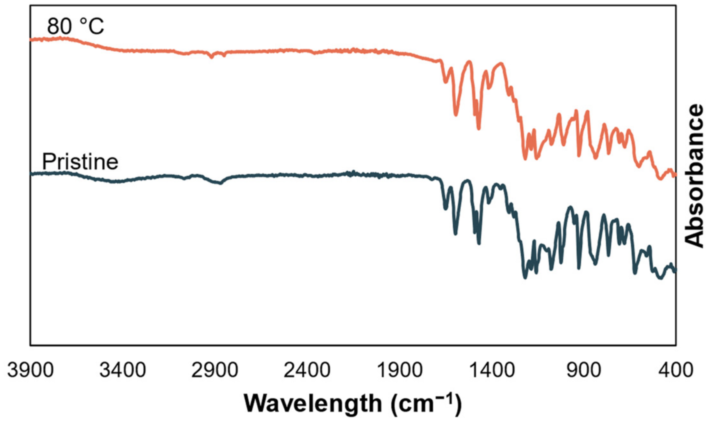

b.

Figure S13: ATR-FTIR spectra of E-620-PE after 1 week 1M KOH treatment at different temperatures (a) and 1 week 1M H_2SO_4 treatment at different temperatures (b).

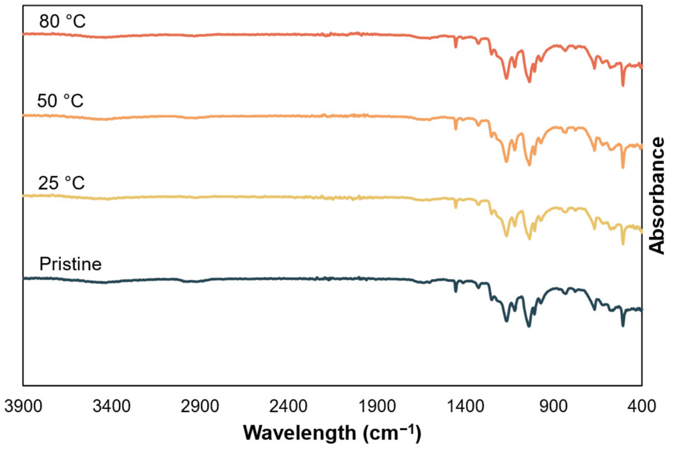

a.

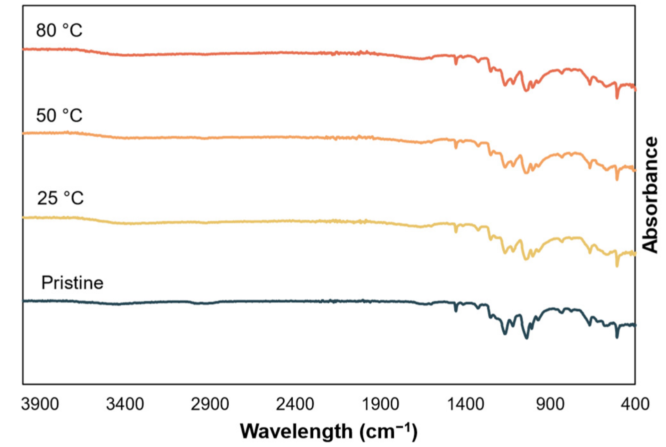

b.

Figure S14: ATR-FTIR spectra of CMVN after 1 week 1M KOH treatment at different temperatures (a) and after 1 week 1M H_2SO_4 treatment at different temperatures plus 42.5 weeks at room temperature (b).

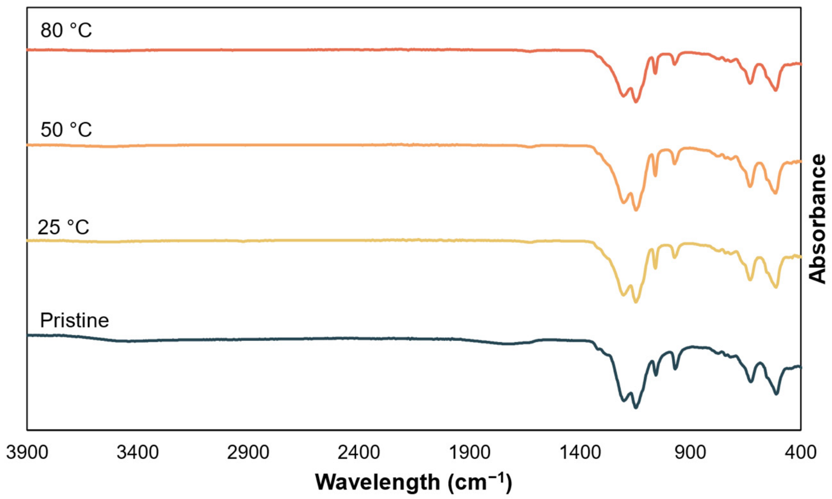

a.

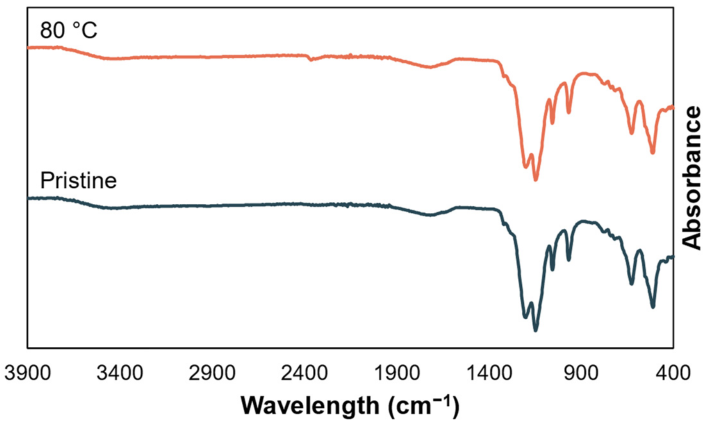

b.

Figure S15: ATR-FTIR spectra of E98-05S after 1 week 1M KOH treatment at different temperatures (a) and 1 week 1M H_2SO_4 treatment at different temperatures (b).

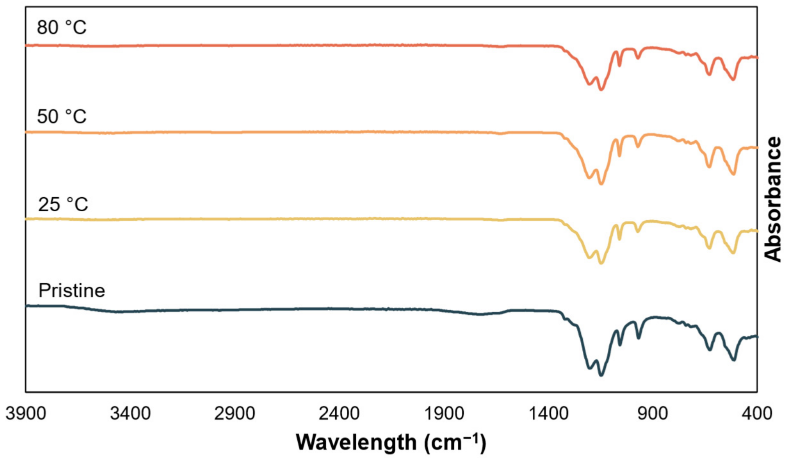

a.

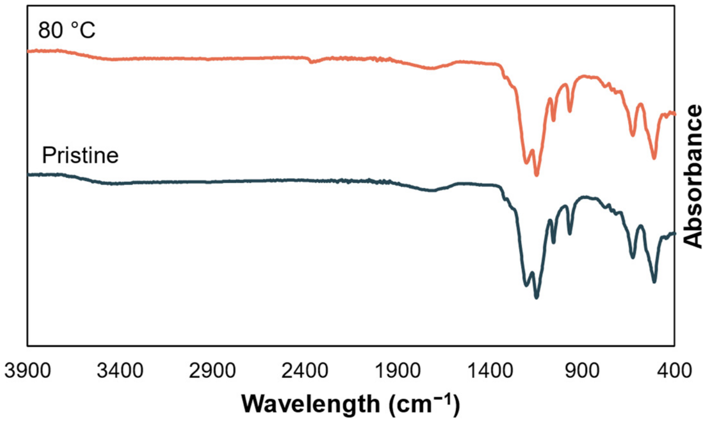

b.

Figure S16: ATR-FTIR spectra of E98-05 after 1 week 1M KOH treatment at different temperatures (a) and 1 week 1M H_2SO_4 treatment at different temperatures (b).

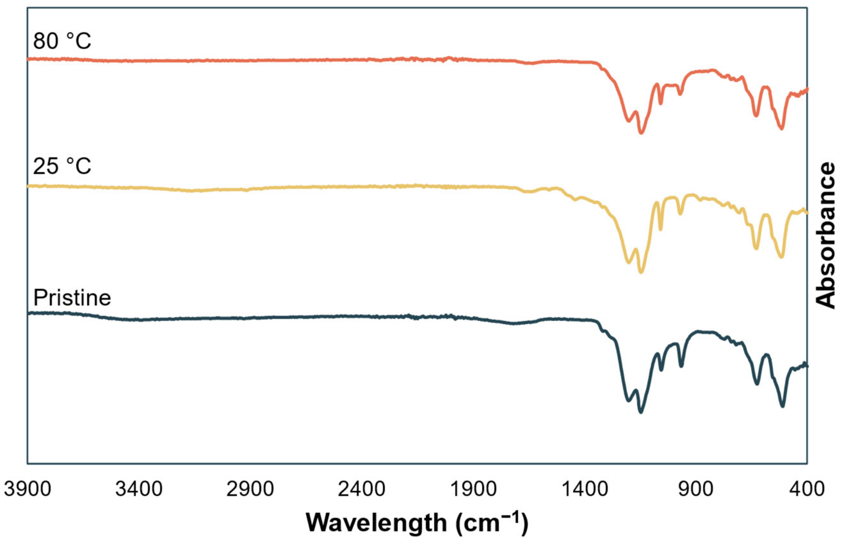

a.

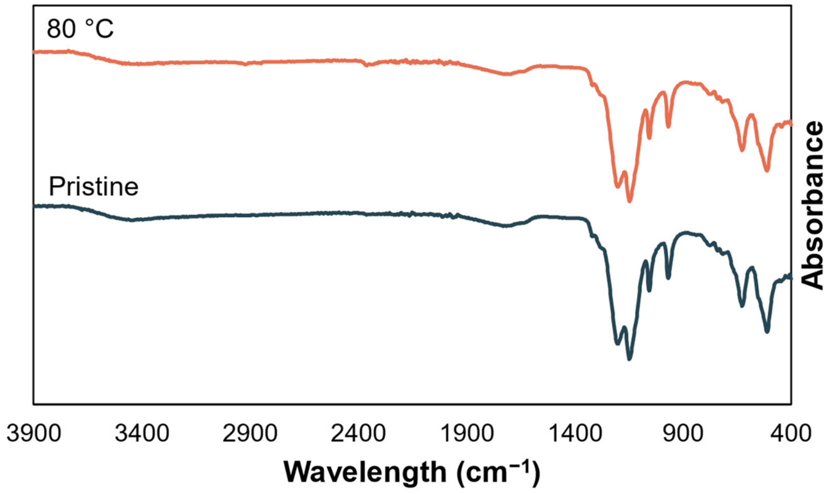

b.

Figure S17: ATR-FTIR spectra of E98-09S after 1 week 1M KOH treatment at different temperatures (a) and 1 week 1M H_2SO_4 treatment at different temperatures (b).

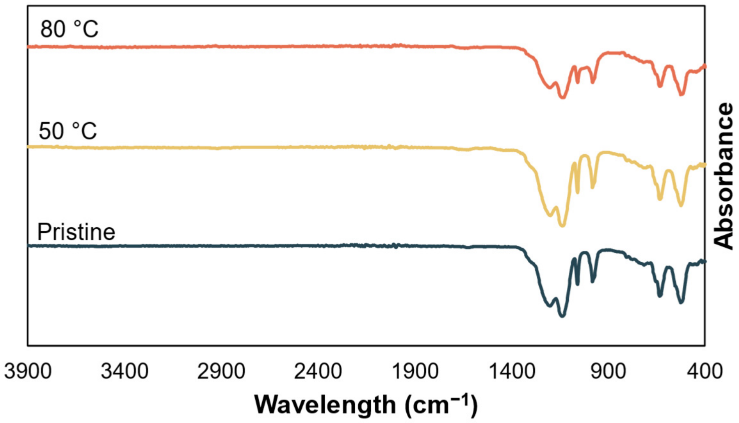

a.

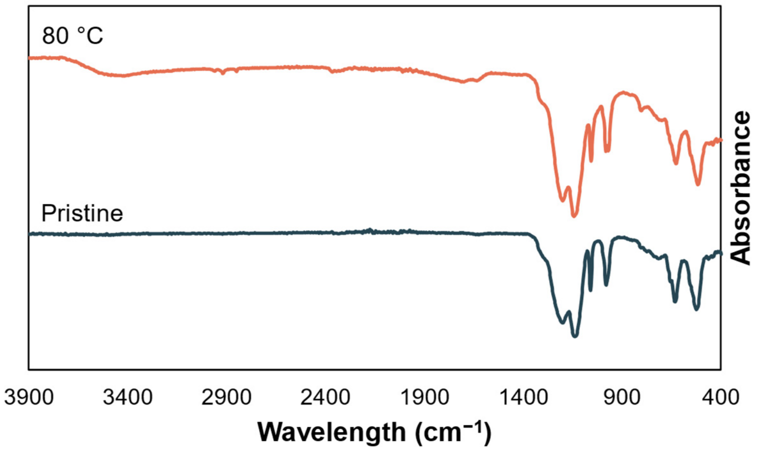

b.

Figure S18: ATR-FTIR spectra of Sx-050DK after 1 week 1M KOH treatment at different temperatures (a) and 1 week 1M H_2SO_4 treatment at different temperatures (b).

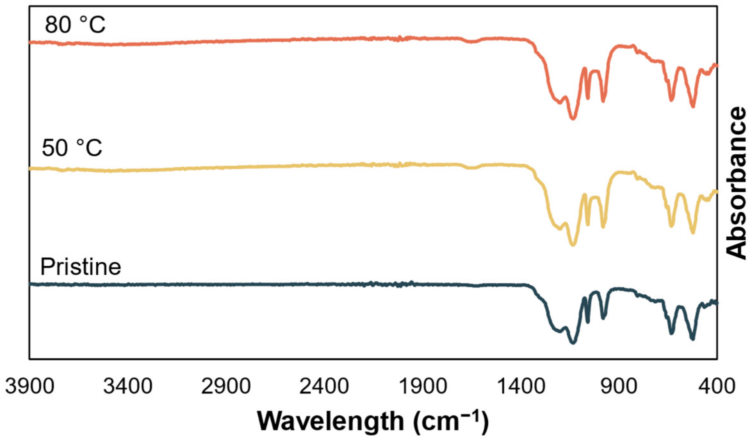

a.

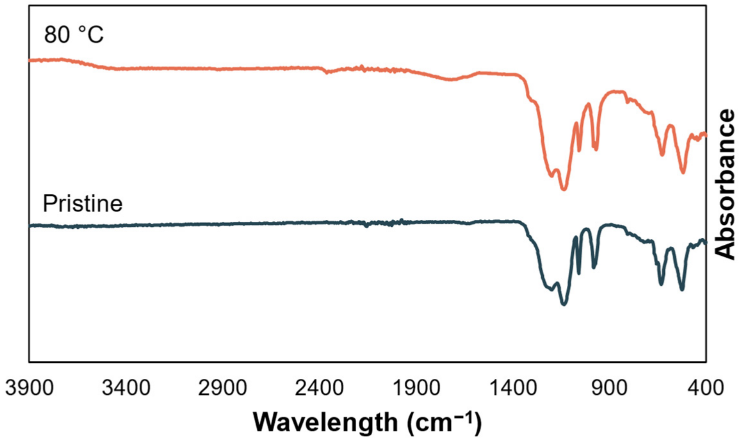

b.

Figure S19: ATR-FTIR spectra of Sx-053DK after 1 week 1M KOH treatment at different temperatures (a) and 1 week 1M H_2SO_4 treatment at different temperatures (b).

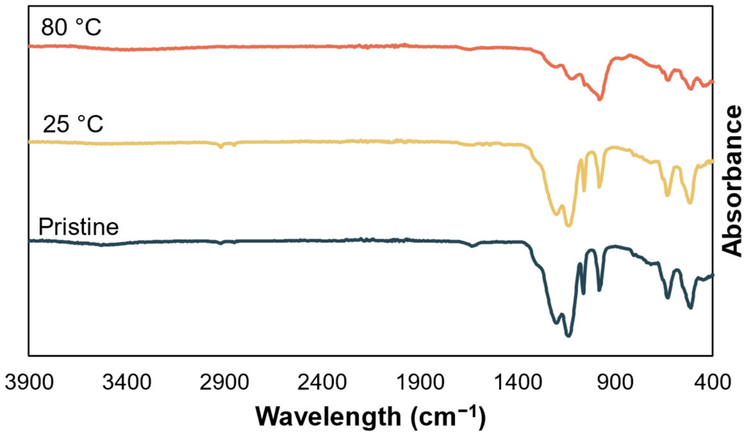

a.

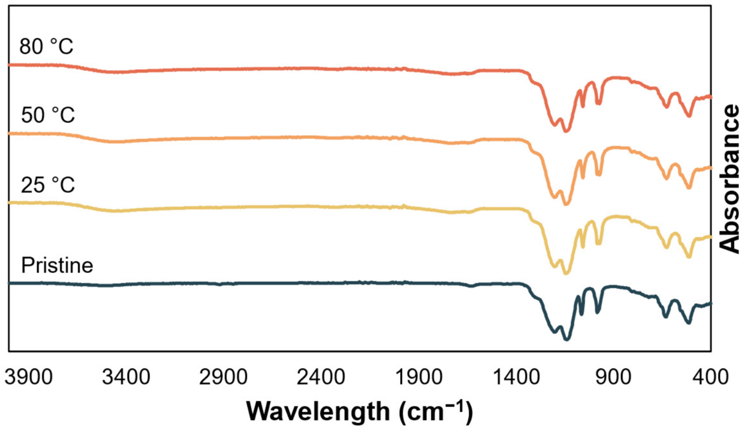

b.

Figure S20: ATR-FTIR spectra of S-2301WN after 1 week 1M KOH treatment at different temperatures (a) and 1 week 1M H_2SO_4 treatment at different temperatures plus 42.5 weeks at room temperature (b).

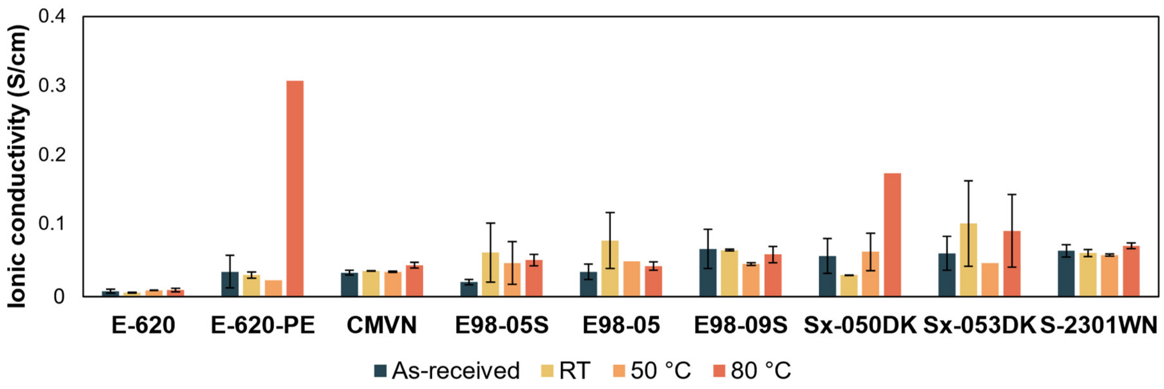

Figure S21: Ionic conductivities before and after storage at RT, 50 °C and 80 °C in 1M H_2SO_4 for 1 week.

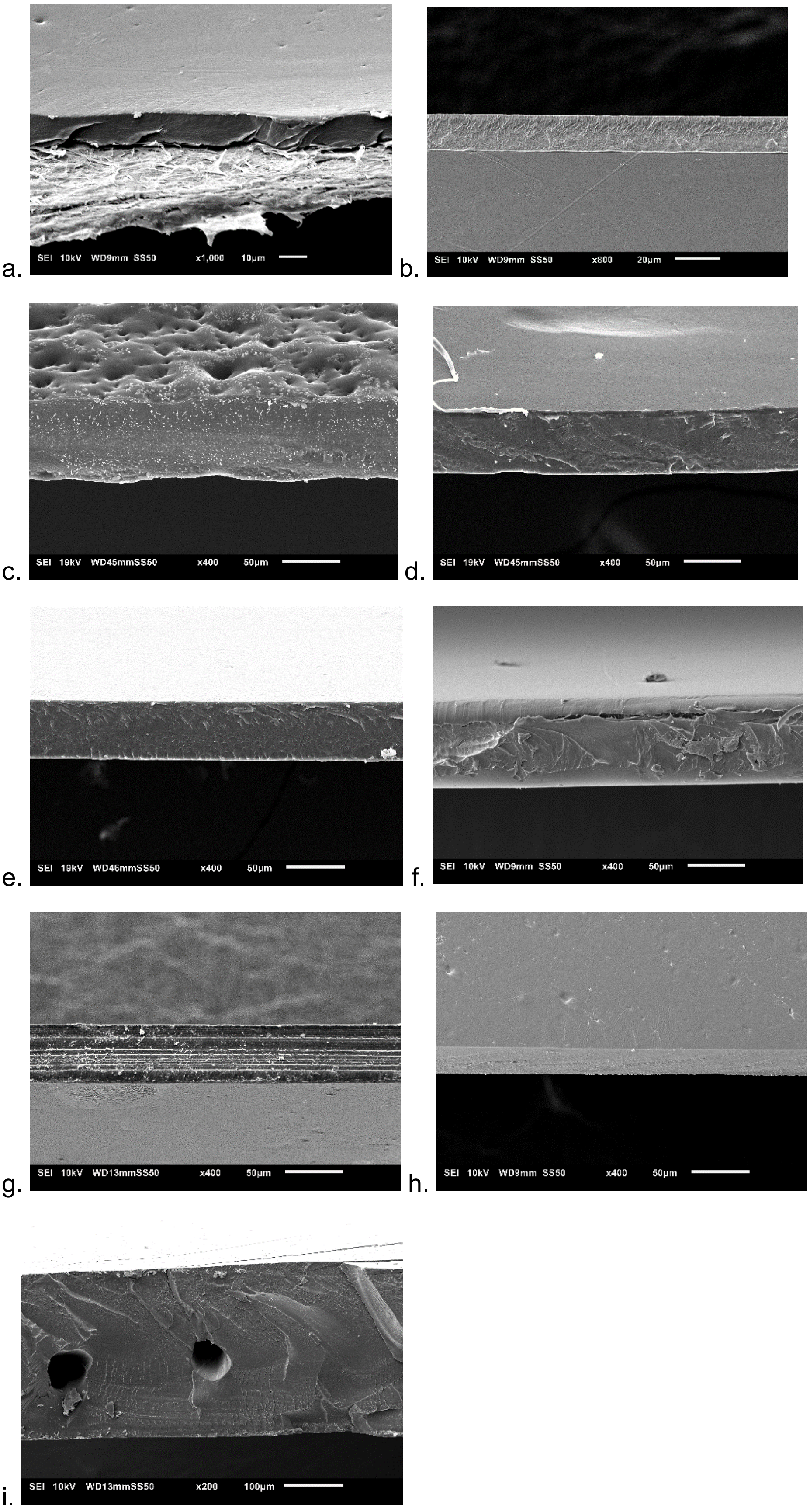

Figure S22: SEM pictures of E-620 (a), E-620-PE (b), CMVN (c), E98-05S (d), E98-05 (e), E98-09S (f), Sx-050DK (g), Sx-053DK (h), S-2301WN (i).
